# Achieving High Specific Strength via Multiple Strengthening Mechanisms in an Fe-Mn-Al-C-Ni-Cr Lightweight Steel

**DOI:** 10.3390/ma18174023

**Published:** 2025-08-28

**Authors:** Rui Bai, Ying Li, Yunfei Du, Yaqin Zhang, Xiuli He, Hongyu Liang

**Affiliations:** 1 Department of Mechanical Engineering, Taiyuan Institute of Technology, Taiyuan 030008, China; duyunfei@tit.edu.cn (Y.D.); zhangyaqin2018tg@163.com (Y.Z.); hexiuli@tit.edu.cn (X.H.); lhy20002025@163.com (H.L.); 2 Shanxi Provincial Inspection and Testing Center, Taiyuan 030006, China; 404315425@163.com

**Keywords:** lightweight steels, B2 precipitates, ultrafine grains, high specific strength, strengthening mechanisms

## Abstract

The development of lightweight steels with high specific strength is critical for automotive applications and energy savings. This study aimed to develop a high-performance lightweight steel with high specific strength by designing an alloy composition and optimizing thermomechanical processing. A novel Fe-28.6Mn-10.2Al-1.1C-3.2Ni-3.9Cr (wt.%) steel was investigated, focusing on microstructural evolution, mechanical properties, and strengthening mechanisms. The steel was processed through hot-rolling, solution treatment, cold-rolling, and subsequent annealing. Microstructural characterization revealed a dual-phase matrix of austenite and ferrite (6.8 vol.%), with B2 precipitates distributed at the grain boundaries and within the austenite matrix, alongside nanoscale κ-carbides (<10 nm). Short-time annealing resulted in the finer austenite grains (~1.1 μm) and the higher volume fraction (5.0%) of intragranular B2 precipitates with a smaller size (~0.18 μm), while long-time annealing promoted the coarsening of austenite grains (~1.6 μm) and the growth of intergranular B2 particles (~0.9 μm). This steel achieved yield strengths of 1130~1218 MPa and tensile strengths of 1360~1397 MPa through multiple strengthening mechanisms, including solid solution strengthening, grain boundary strengthening, dislocation strengthening, and precipitation strengthening.

## 1. Introduction

Fe-Mn-Al-C lightweight steels exhibit significant application potential in fields such as automotive and aerospace, due to their excellent combination of properties of low density, high strength, and good ductility [1,2,3]. Performance improvement in these steels relies on the design of alloy compositions [4,5] and the optimization of thermomechanical processing [6,7,8]. The addition of Al not only reduces density (approximately 1.3% reduction per 1 wt.% Al) but also collaborates with C to promote the precipitation of κ-carbides ((Fe, Mn)_3_AlC) [9,10]. Mn acts as an austenite stabilizer, suppressing the formation of brittle phases, with its content (15~30 wt.%) being crucial for maintaining austenite stability [11,12]. Furthermore, the incorporation of Ni represents a key strengthening strategy that facilitates the formation of the B2 phase (NiAl-type intermetallic compound). This phase enhances strength through the Orowan mechanism while simultaneously improving toughness by controlling its morphology and distribution [13,14].

In Fe-Mn-Al-C-Ni lightweight steels, the addition of Ni is critical for regulating the formation of the B2 phase, significantly enhancing both its strength and strain hardening ability [15,16]. Studies have demonstrated that in Fe-(15–21)Mn-(8–10)Al-(0.8–1)C-5Ni (wt.%) steels, the B2 phase exists in various forms, including elongated band-like particles (1~5 μm in width) along the rolling direction, polygonal particles at grain boundaries (400 nm to 2 μm), and fine particles within austenite grains (50~500 nm) [13,17,18,19]. The non-shearing characteristics of the B2 phase effectively impede dislocation motion, thereby enhancing strain hardening. However, coarse band-like B2 phases can initiate voids and microcracks at phase interfaces, negatively impacting ductility [18,19]. By optimizing composition and processing to eliminate band-like B2 phases, it is possible to maintain high strength while improving formability. For instance, Fe-16Mn-8Al-1C-5Ni (wt.%) steel showed significant improvements in formability due to the uniform distribution of B2 particles [18].

Mechanical deformation and heat treatment are critical for controlling the distribution and size of the B2 phase, which directly affects the final properties of lightweight steel [20,21]. Post-cold-rolling annealing (above 800 °C) is essential for managing the morphology of the B2 phase: lower temperatures or shorter annealing times tend to form partial recrystallized structures, where B2 phases nucleate mainly at dislocations in unrecrystallized areas or at austenite grain boundaries, resulting in uneven distribution [13,19,22]. Conversely, higher temperatures or longer annealing durations promote complete recrystallization, which may lead to the coarsening of B2 phases at grain boundaries (size ≥ 1 μm) [23,24,25]. In recent years, dual nanoprecipitation strengthening strategies (B2 phase + κ-carbides) have further promoted the performance of these steels. For example, the synergy between these two precipitates allowed Fe-Mn-Al-C-Ni steels to achieve ultra-high tensile strength (1.6~1.7 GPa) alongside good ductility (13~39.3%) [19,22,23,26,27]. Furthermore, regulating the ratio of nanoscale κ’-carbides to the B2 phase through aging can balance precipitation hardening, although care must be taken to avoid the embrittlement caused by the coarsening of κ’-carbides and B2 precipitates with an increase in aging time [23,28,29,30].

Previous research [31] identified that the addition of Cr (2.5~5 wt.%) to the Fe-25Mn-10Al-1.2C (wt.%) steel can inhibit the growth of austenite grains during recrystallization. In other studies on B2-strengthened lightweight steels, achieving the formation and stabilization of the B2 phase typically required the addition of about 5 wt.% Ni. Therefore, this study successfully developed a high-strength lightweight steel containing a low Ni content (3.2 wt.%) and an appropriate Cr content (3.9 wt.%) and optimized the precipitation behavior of B2 precipitates and the recrystallization behavior of grains through hot-rolling, solid solution treatment, cold-rolling, and annealing. This approach utilizes the synergistic effects of multiple strengthening mechanisms, demonstrating a significant enhancement in mechanical properties and providing new insights for the development of lightweight high-strength steels.

## 2. Materials and Methods

**Figure 1:**
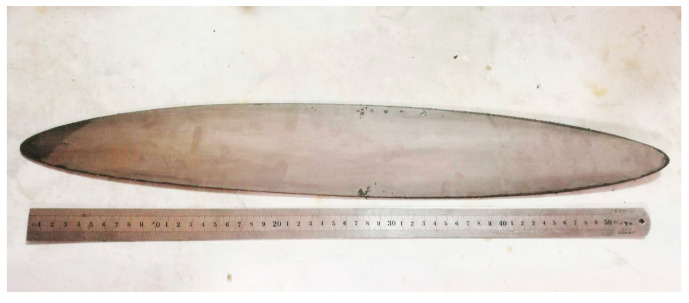
Real photograph of the cold-rolled sheet.

A 20 kg lightweight steel ingot was prepared by vacuum induction melting. The chemical composition of the steel obtained by chemical analysis is Fe-28.6Mn-10.2Al-1.1C-3.2Ni-3.9Cr (wt.%). The density measured through the Archimedes method was 6.6 g·cm^−3^. The ingot was homogenized at 1200 °C for 4 h. The hot- and cold-rolling processes were conducted on a two-roll mill (with a maximum strip width of 400 mm). A 30 mm thick block was cut from the ingot and then hot-rolled at a starting temperature of 1100 °C to produce an 8 mm thick plate. The hot-rolled plate was subsequently solid-solution-treated at 900~1100 °C for 1 h. Then the plate was cold-rolled to a final thickness of 2 mm with a total reduction rate of 80%. The real photograph of the cold-rolled sheet is shown in Figure 1. The cold-rolled sheet was annealed at 900 °C for different times and immediately quenched in water. For convenience, the sample solution treated at 1100 °C is referred to as ST1100, and the samples annealed at 900 °C for 3 min and 15 min are referred to as A900-3 and A900-15, respectively.

Phase identification was conducted by X-ray diffraction (XRD) using Rigaku D/max 2550 (Rigaku Corporation, Tokyo, Japan) in a scanning 2θ range of 20°~100° at a scanning rate of 2°/min and a step size of 0.02°. The microstructures of the samples were studied by a Zeiss Axio imager optical microscope (OM, Carl Zeiss AG, Oberkochen, Germany), Zeiss Supra55 scanning electron microscope (SEM, Carl Zeiss AG, Oberkochen, Germany), electron backscatter diffraction (EBSD, Oxford Instruments, Oxford, UK), and Tecnai G2 F20 transmission electron microscopy (TEM, FEI, Hillsboro, OR, USA). The EBSD samples were prepared by electropolishing in 90% ethanol and 10% perchloric acid solution, and measurement was carried out under an acceleration voltage of 20 kV. TEM thin films were prepared by double-jet electropolishing in 95% acetic acid + 5% perchloric acid solution at 20 V and −25 °C, and measurement was carried out at a working voltage of 200 kV.

Tensile samples, with a length, width, and thickness of 55 mm, 5 mm, and 2 mm, respectively, were cut along the rolling direction. Tensile tests were conducted at a strain rate of 10^−3^ s^−1^ using a CMT4105 electronic universal testing machine (Sansi Testing Instrument Ltd., Shenzhen, China). The volume fraction of recrystallization and the average size of recrystallized grains were calculated by EBSD. The volume fraction and the average size of B2 particles at grain boundaries and within the austenite matrix were determined by EBSD and SEM micrographs.

## 3. Results

### 3.1. Microstructure of As-Solutionized Sample After Hot-Rolling

**Figure 2:**
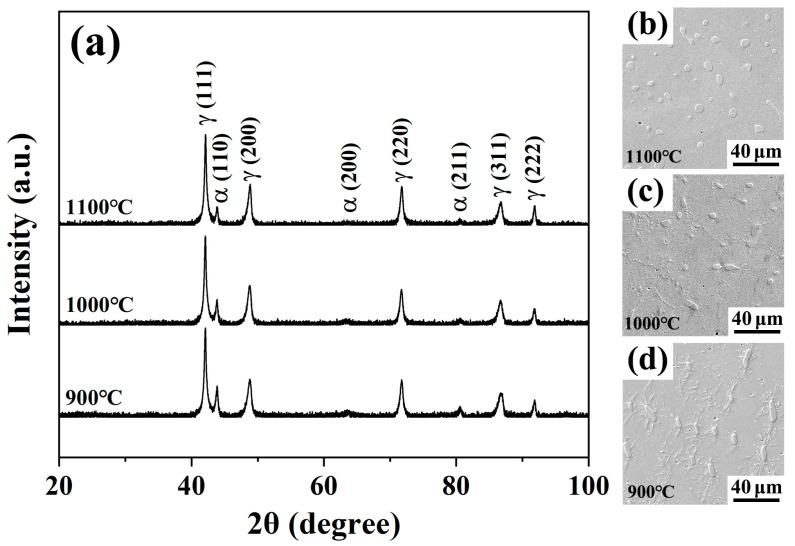
(**a**) XRD patterns and (**b**–**d**) SEM micrographs of the steel after solid solution treatment at 1100 °C, 1000 °C, and 900 °C for 1 h.

Figure 2 presents the XRD patterns and SEM microstructures of the hot-rolled sheets after solution heat treatment at different temperatures. In Figure 2a, all three curves exhibit characteristic peaks of austenite (γ) and ferrite (α), indicating that the matrix maintains a γ + α dual-phase structure after solution treatment at temperatures ranging from 900 °C to 1100 °C. As the solution temperature decreases, the intensity of α-phase characteristic peaks relatively increases, while that of γ-phase peaks decreases, suggesting an increase in the proportion of the α-phase. Notably, no diffraction peaks corresponding to κ-carbides were detected, implying their complete dissolution during the high-temperature treatment. As shown in Figure 2b, after solution treatment at 1100 °C, the ferrite phases were dispersed in the austenite matrix with a volume fraction of approximately 6.8%, indicating that high-temperature solution treatment enables the ferrite phase to distribute uniformly in the austenite matrix. Some fine precipitated particles began to form along austenite grain boundaries in the microstructure after solution treatment at 1000 °C (Figure 2c). With the number of precipitated particles further increasing after solution treatment at 900 °C (Figure 2d), needle-shaped and fine strip-shaped precipitates appeared, which were distributed around ferrite. This suggests that these precipitates may grow with a specific crystallographic orientation relative to ferrite. Due to their small size and low content, the diffraction peaks of these precipitated phases were not identified in the XRD patterns.

In a single-phase austenitic matrix, high-temperature treatment promoted the dissolution of κ-carbides and induced the uniform precipitation of the B2 phase as nanoscale particles during the subsequent annealing [22]. In Fe-28Mn-11Al-1.1C-5Ni (wt.%) steel, after annealing at 900 °C, κ-carbides completely dissolve, allowing the B2 phase to facilitate heterogeneous nucleation through the vacancies created by this dissolution [32]. Conversely, in a duplex microstructure consisting of both austenite and ferrite, the distribution of the B2 phase after annealing was influenced by the interphase boundaries. Ferrite, serving as an Al-rich phase, provided the preferential precipitation of the B2 phase at the austenite–ferrite interfaces, resulting in the formation of polygonal B2 particles with a size of 1~3 μm [33]. In contrast, the smaller B2 particles, with sizes below 500 nm, were dispersed within the austenite grains. The study of Fe-21Mn-9Al-1C-8Ni (wt.%) steel [34] indicated that after annealing at 900 °C, the volume fraction of the B2 phase in the ferrite region (15~20%) was significantly greater than that in austenite (5~8%). Additionally, the presence of the B2 phase at the interfaces can inhibit the coarsening of ferrite, thereby stabilizing the ratio of the two matrix phases.

### 3.2. The Microstructure of As-Annealed Samples After Cold-Rolling

**Figure 3:**
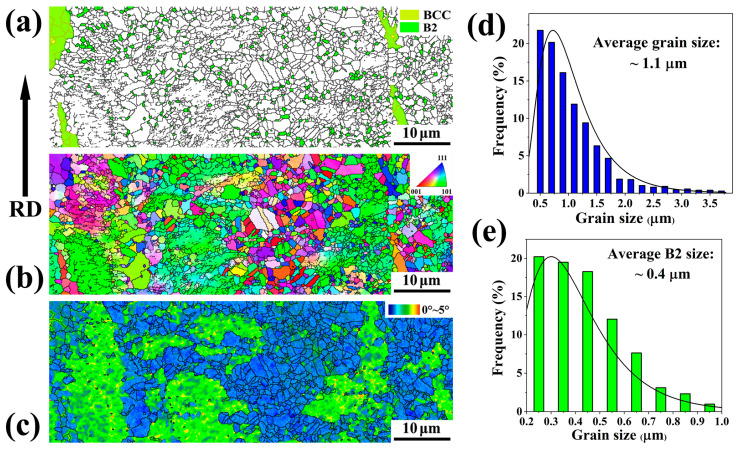
EBSD characterization and analysis of A900-3 sample. (**a**) Phase map; (**b**) IPF map; (**c**) KAM map; (**d**) distribution of austenite grain size; (**e**) distribution of B2 particle size.

**Figure 4:**
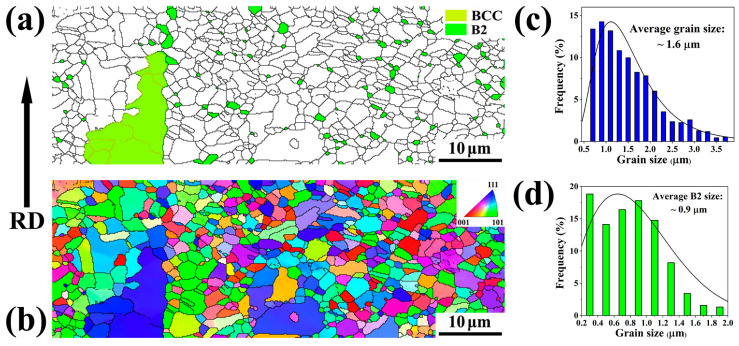
EBSD characterization and analysis of A900-15 sample. (**a**) Phase map; (**b**) IPF map; (**c**) distribution of austenite grain size; (**d**) distribution of B2 particle size.

Figure 3 and Figure 4 display the EBSD characterization and analysis of the two samples. The EBSD phase distribution map illustrates that the B2 phase (green color) exists as fine particles dispersed within the austenitic matrix (white color), along with ferrite phases (yellow color), in Figure 3a and Figure 4a. The inverse pole figure (IPF) map and the Kernel Average Misorientation (KAM) map are used to characterize the crystal orientation distribution and the degree of grain misorientation. The calculated average KAM values of austenite can be used to distinguish recrystallized grains and unrecrystallized grains. The grains with low KAM values (less than 1°) are considered recrystallized grains, while the grains with high KAM values (1°–5°) are unrecrystallized grains. Specifically, for the A900-3 sample, the volume fraction of austenite was measured at 51.7% with an average recrystallized grain size of 1.1 μm. The B2 phase at the grain boundaries (GB) occupies a 4.5% volume fraction, with an average particle size of 0.4 μm, while the volume fraction of B2 within the matrix is 5.0%, with an approximate size of 0.18 μm. In comparison, for the A900-15 sample, the volume fraction of austenite increases to 79.3%, accompanied by an average grain size of 1.6 μm. The volume fraction of B2 at the grain boundaries rises to 5.3%, with an increased average size of 0.9 μm. In the matrix, the B2 volume fraction is reduced to 3.6%, revealing a particle size of 0.33 μm. The observations also indicate the refinement of ferrite grains, which measure approximately 2.7 μm and 3.2 μm in size. The results of the size and volume fraction measurements of austenite and B2 particles are summarized in Table 1.

In comparison, under the same conditions (annealing at 900 °C for 15 min), Fe-24Mn-11Al-0.9C-5Ni steel exhibited a bimodal grain distribution in its austenite phase, characterized by ultrafine grains (less than 1 μm) and coarser grains (approximately 1.85 μm), with volume fractions of 54% and 46%, respectively [25]. The overall volume fraction of the B2 phase was approximately 35%. The B2 precipitates at the grain boundaries were polygonal particles with sizes around 0.6 μm and a volume fraction of about 10% [25]. The microstructure of Fe-21Mn-10Al-1C-5Ni (wt.%) steel exhibited fully recrystallized austenite grains with sizes averaging around 1.8 μm and a volume fraction of approximately 80%. The polygonal B2 particles at the grain boundaries were measured to be about 0.45 μm in size, comprising a volume fraction of around 14% [19]. A comparative analysis of these steels indicates that after recrystallization under the same annealing conditions, the size of austenite grains is significantly influenced by the alloy composition, resulting in notable differences in the distribution, volume fractions, and dimensions of both the boundary and intragranular B2 phases.

The results demonstrate that short-time annealing leads to a lower proportion of recrystallized austenite with smaller grain sizes, whereas long-time annealing allows for more complete recrystallization and a higher proportion of coarser austenitic grains. Furthermore, extended annealing conditions provide more nucleation sites for the formation of B2 precipitates at the grain boundaries, facilitating their growth. During the initial stage of annealing, the nucleation rate of B2 precipitates is higher, but with an increase in annealing time, certain B2 precipitates within the matrix coarsen and migrate towards the grain boundaries. This process results in a decrease in the volume fraction of B2 within the matrix while concurrently increasing their size.

**Figure 5:**
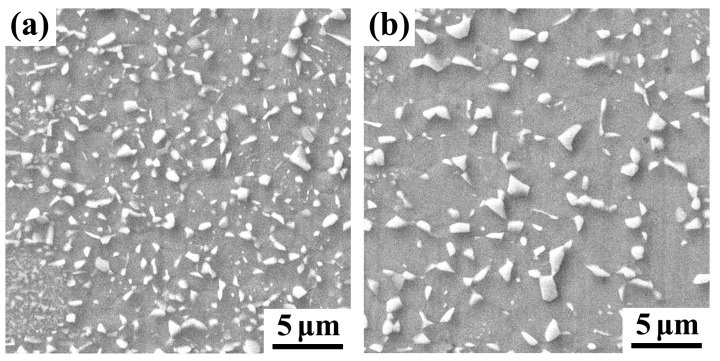
SEM micrograph showing morphology of B2 precipitates. (**a**) A900-3 sample; (**b**) A900-15 sample.

Figure 5a displays the SEM microstructure of the A900-3 sample. It can be observed from the micrograph that the B2 precipitated particles are relatively small. Most of the particles are sub-micron in size, indicating that the precipitated phases did not fully grow within the short annealing time. A relatively large number of precipitated phases are dispersed throughout the austenite matrix. Figure 5b shows the SEM microstructure of the A900-15 sample, where the B2 precipitated particles grew significantly. Some particles approach or exceed 1 μm in size, demonstrating that the longer annealing time allowed sufficient time for precipitate aggregation and coarsening. The number of precipitated phases decreased, and their distribution became relatively sparse. This is because as the particles grew, some small particles merged into large ones, resulting in a reduction in the number. Particle size measurements from the images show that in the A900-3 sample, the B2 precipitates have an average equivalent diameter of 0.3~0.5 μm, while in the A900-15 sample, the average size increases to 0.8~1.2 μm. The short annealing time limits atomic diffusion distances, resulting in smaller particles, whereas the extended annealing provides adequate energy for the aggregation and growth of B2 precipitates through atomic diffusion, leading to a significant increase in particle size.

**Figure 6:**
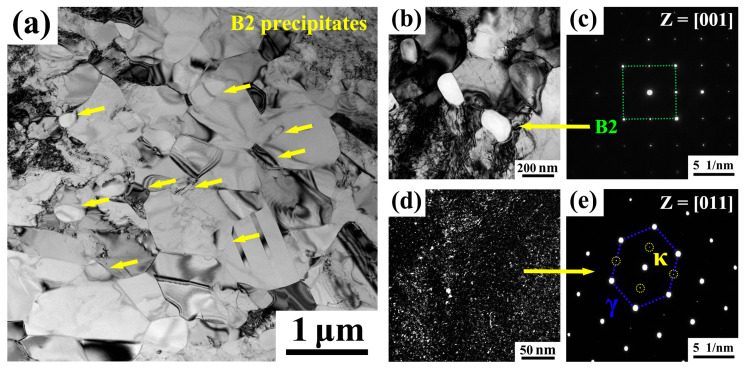
TEM microstructural morphology of A900-3 sample. (**a**) Bright-field image showing the recrystallized austenite grain and B2 precipitates (as marked by yellow arrows). (**b**,**c**) Bright-field image and corresponding SAED pattern of B2 particles. (**d**,**e**) Dark-field image and corresponding SAED pattern of κ-carbides.

Figure 6 presents the TEM microstructural morphology of the A900-3 sample. In Figure 6a, a distinct grain structure is observed, where the austenite grains in the matrix vary in size. The microstructure contains dislocations and twins, which represent defects retained from the cold-rolling and annealing processes. Bright white particles indicated by yellow arrows correspond to B2 precipitates, showing the intergranular distribution along grain boundaries. These precipitates exhibit a granular morphology with relatively uniform dispersion. Figure 6b illustrates the morphology of the B2 precipitates, which appeared as relatively regular granules with a size of approximately 200 nm. The spacing between B2 particles forces dislocations to bypass them during movement, generating significant resistance and thereby enhancing strength. The selected area electron diffraction (SAED) pattern in Figure 6c, taken along the [001] zone axis, confirms the body-centered cubic structure of the B2 phase through its characteristic diffraction spots. Figure 6d displays the morphology of κ-carbides precipitated within the austenite. These carbides existed as extremely fine particles, uniformly distributed in the austenite matrix with a size smaller than 10 nm. Such fine, uniformly distributed nanoscale carbides contributed to strengthening and toughening. Figure 6e provides the SAED pattern of κ-carbides along the [011] zone axis, revealing coherent diffraction spots from both the carbides and austenite matrix.

### 3.3. Mechanical Properties and Strain Hardening Behavior

**Figure 7:**
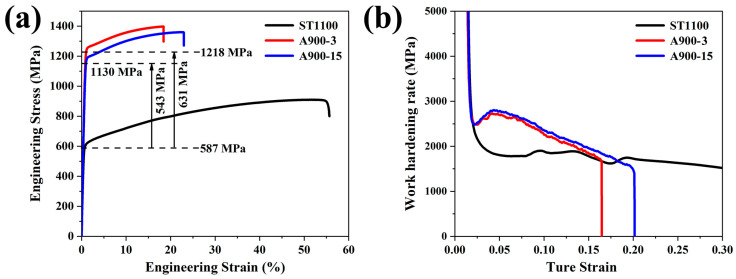
Room-temperature tensile properties of present steel under different conditions. (**a**) Engineering stress–strain curves; (**b**) work hardening rate versus true strain curves.

Figure 7a shows the room-temperature tensile curves of the as-solutionized sample and as-annealed samples of the present steel. The ST1100 sample exhibits a yield strength of 587 MPa, a tensile strength of 909 MPa, and a considerable fracture strain of 54.9%, indicating excellent ductility. In contrast, both the A900-3 and A900-15 samples show significantly higher yield and tensile strengths compared to the ST1100 sample, with yield strengths of approximately 1130 MPa and 1218 MPa, respectively, and tensile strengths of 1360 MPa and 1397 MPa, respectively. However, the fracture strains are relatively lower than that of ST1100, measuring 23.0% and 18.4%, respectively. Compared to the hot-rolled solution-treated state, the cold-rolled annealed state provided a remarkable improvement in yield strength, with A900-3 showing an enhancement of 631 MPa over ST1100, while A900-15 exhibits an increase of 543 MPa.

Figure 7b illustrates the corresponding work hardening rate evolution for the three samples. The ST1100 sample displays a characteristic rapid initial decline followed by sustained yielding and extensive strain hardening. The work hardening rate progressively increases from plastic deformation onset (strain ~0.05), and then a stable work hardening rate (~1500–2000 MPa) is maintained at a strain of 0.05~0.3. Both the A900-3 and A900-15 samples exhibit different behavior: after an initial rapid decrease at a strain below 0.025, the work hardening rates sharply increase to the maximum values around a strain of 0.05 before the subsequent decline, stabilizing near 2000 MPa at ~0.15 strain. Notably, the A900-3 sample, containing a higher fraction of unrecrystallized austenite grains, due to higher dislocation density and poor deformation compatibility, leads to a strain hardening rate that remains slightly lower than that of A900-15. Overall, the hot-rolled and solution-treated sample exhibits low yield strength, a slow decline in the hardening rate, and a low yield-strength-to-tensile-strength ratio. Conversely, the cold-rolled and annealed samples demonstrate high yield strength but exhibit a rapid decrease in the hardening rate and a higher yield-strength-to-tensile-strength ratio.

### 3.4. The Deformation Microstructure

**Figure 8:**
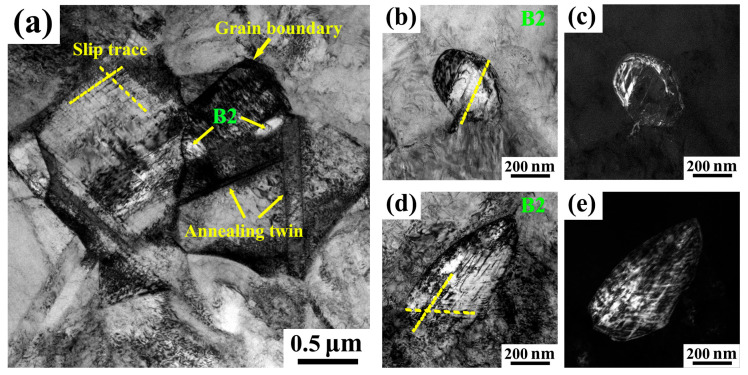
TEM micrographs showing the deformed microstructures of A900-3 sample at strain of 10%. (**a**) Bright-field image of austenitic matrix. (**b**,**d**) Bright-field image showing interaction between B2 particles and dislocations. (**c**,**e**) Corresponding dark-field image of (**b**,**d**). Slip traces in B2 particles (**b**,**d**) are indicated by yellow dashed lines.

The TEM characterization results reveal the tensile deformation features of the A900-3 sample at 10% strain. Figure 8a shows a bright-field image of the austenite matrix, illustrating the overall deformation of the sample. Slip traces, grain boundaries, and annealing twins can be observed within the austenite matrix. These slip traces are identified as the (1$\bar{1}$1) and (11$\bar{1}$) planes. The grain boundaries and annealing twins act as barriers to dislocation movement, causing dislocations to accumulate nearby, thereby enhancing strain hardening capabilities. The interactions between dislocations and grain boundaries or twins are crucial factors contributing to strain hardening. Figure 8b,c show the bright-field image and corresponding dark-field images of the interaction between B2 particles and dislocations, respectively. It can be seen that there is a significant interaction between dislocations and B2 particles. B2 precipitates exhibit non-coherent characteristics, and their interface with the austenite matrix (B2/FCC interface) is a significant barrier to dislocation slip. When dislocation motion encounters B2 particles, bending and entanglement occur around the particles, increasing resistance to dislocation motion and resulting in strain hardening. This interaction between dislocations and B2 particles is considered a key factor in achieving high strain hardening capacity. Figure 8d,e further illustrate the interaction between B2 particles and dislocations. The images show not only pronounced entanglements of dislocations around the B2 particles but also the emergence of slip traces within the B2 particles themselves. This suggests that as deformation increases, some dislocations remain trapped inside the B2 precipitates when passing through them.

The interaction between dislocations and precipitated phases during tensile deformation plays an important role in strain hardening ability [19,23,25]. The interaction mechanism of dislocations with dual nanoprecipitate phases is different, as observed in Fe-29Mn-9Al-1C-5Ni (wt.%) steel [23]. B2 precipitates facilitate dislocation accumulation around them due to a bypassing mechanism, forming loops, while κ’-carbides can be sheared by dislocations, allowing dislocations to both accumulate around and penetrate through the interior of the particles. In contrast, the evolution of the deformed microstructure observed in these steels [19,25] indicates that at low strains, planar dislocation slip occurs on the {111} planes with dislocations largely migrating within the austenite matrix and a lower dislocation density observed within the B2 phase. As the strain increases, there is a substantial rise in dislocation density at the austenite/B2 interface, where dislocations can be emitted from the interface and traverse through small B2 particles. At high strains, dense dislocation walls form within the slip bands, resulting in the severe entanglement of dislocations at the interfaces, with a significant accumulation of dislocations also occurring within the B2 particles. These findings indicate that the B2 phase becomes actively involved in plastic deformation during the later stages of deformation.

## 4. Discussion

### 4.1. The Role of Alloying Elements

In this developed steel, the relatively high Mn content of 28.6 wt.% can effectively inhibit the formation of ferrite, enabling the steel to have an austenitic matrix at room temperature and thus improving its strength and toughness. Compared with other steels with low Mn content [17,24], the high Mn content enhances the stability of austenite and reduces the occurrence of brittle phases and also affects the precipitation and distribution of the B2 phase [24]. The C alloying element is mainly used to stabilize austenite and form carbide strengthening phases. The C content in Ni-containing lightweight steels with high strength generally ranges from 0.8 to 1.2 wt.% [17,19,22,26]. An appropriate amount of C can increase the strength of the steel, but an excessively high C content will reduce its plasticity [4,5]. The Al alloying element plays an important role in lightweight steels. It not only reduces the density of the steel but also promotes the precipitation of the B2 phase and κ-carbides. The Al content of 10.2 wt.% can effectively reduce the density while providing sufficient Al for the formation of strengthening phases. The Ni alloying element in lightweight steels mainly promotes the formation of the B2 phase. The Ni content of 3.2 wt.% in this steel is lower than that in most Ni-containing lightweight steels, leading to a reduction in cost. The Cr alloying element is a unique additive element. It can significantly improve the corrosion resistance of the steel [35] and also refines grains and enhances the strength and hardness of the steel [31,36].

At a relatively high solution temperature of 1100 °C, solute atoms such as Ni and Al are fully dissolved in the austenitic matrix, forming a supersaturated solid solution. This is because the high temperature provides sufficient energy for solute atoms to overcome diffusion resistance and distribute uniformly in austenite. As the solution temperature decreases to 900 °C, the solubility of solute atoms (Ni, Al) in austenite decreases. According to the relationship between solubility and temperature, austenite cannot accommodate such a large number of Ni and Al atoms at lower temperatures, resulting in supersaturation. This supersaturation serves as an important driving force for the precipitation of the B2 phase. When the two elements reach a 1:1 stoichiometric ratio in local regions, they nucleate and grow with the ordered body-centered cubic structure of the B2 phase [15]. During hot-rolling, the Ni and Al dissolved in the austenitic matrix diffuse and aggregate in the stages of dynamic recovery and recrystallization, preferentially forming the B2 phase in ferrite regions and at austenite–ferrite interfaces, with a volume fraction reaching 15~20% [24]. Moreover, the enrichment of Al in ferrite further promotes the formation of the B2 phase. On the other hand, during hot-rolling and annealing, the existing κ-carbides undergo partial or complete dissolution at high temperatures, releasing a large amount of Al. This Al further combines with Ni in the matrix, serving as a supplementary solute source for the B2 phase [13].

### 4.2. Precipitation of B2 Phase and Recrystallization Behavior

**Figure 9:**
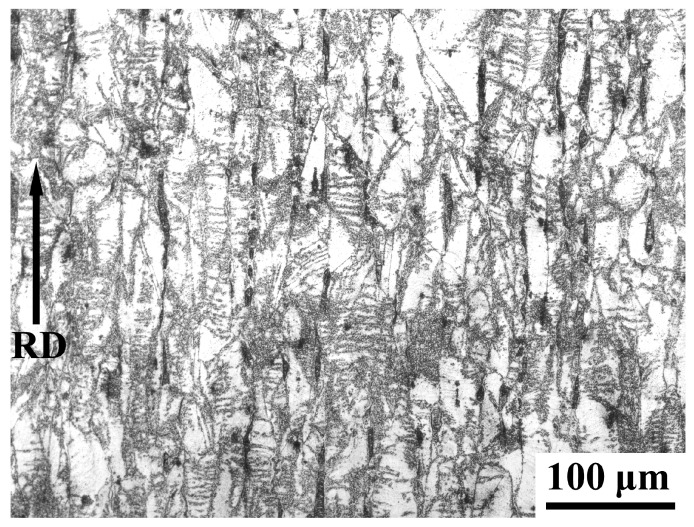
OM micrograph showing the deformed microstructure of cold-rolling sheet.

Figure 9 shows the deformed microstructure of the cold-rolling sheet. The austenite grains were elongated along the rolling direction. This elongation is due to the slip and rearrangement of dislocations within the austenite grains, which makes the originally equiaxed or irregularly shaped grains gradually transform into a fibrous structure. The black elongated strips are ferrite grains. Similarly to austenite grains, ferrite grains were also elongated along the rolling direction. During the cold-rolling process, under the action of external forces, the austenite grains were subjected to severe plastic deformation. This severe deformation also led to the formation of shear bands, microbands, deformation twins, and other dislocation substructures in austenite grains, making it difficult to even distinguish the boundaries of the original grains. During the annealing process after cold-rolling, the formation of the B2 phase is closely related to defects induced by deformation stored energy (dislocations, subgrain boundaries), and its morphology and distribution are significantly affected by recrystallization. The high-density dislocations and deformed substructures introduced by cold-rolling provide numerous nucleation sites for the B2 phase. In the early stage of annealing (partial recrystallization stage), the B2 phase preferentially precipitates along dislocation lines or subgrain boundaries in unrecrystallized regions, forming fine particles with sizes of 50–300 nm [15,18]. When the annealing temperature increases or the time is extended (full recrystallization stage), recrystallized austenite grain boundaries become new nucleation centers, where the B2 phase grows into polygonal particles with sizes of 350–700 nm [18,19].

The deformation energy stored during cold-rolling provides the driving force for recrystallization. The recrystallization behavior during annealing mainly manifests as recovery, recrystallization, and grain growth, and its kinetics are significantly influenced by the precipitation of the B2 phase [15,24]. The B2 phase has a dual effect on recrystallization. Fine B2 particles (<300 nm) can delay the initiation of recrystallization by pinning dislocations and subgrain boundaries. For example, in Fe-16Mn-10Al-0.86C-5Ni (wt.%) steel [19], during the partial recrystallization stage, fine intragranular B2 particles inhibit dislocation migration, increasing the recrystallization temperature by approximately 50 °C. In contrast, coarsened B2 phases (>500 nm) have a weakened inhibitory effect on recrystallization and can even act as nucleation cores for recrystallization, accelerating grain growth. When B2 phases aggregate at recrystallized grain boundaries, the coarsening rate of austenitic grains increases by 20~30% [24]. Some B2 particles can act as heterogeneous nucleation cores for recrystallization, promoting the formation of recrystallized grains. During the annealing of Fe-24Mn-11Al-0.9C-5Ni (wt.%) steel [25], strain concentration around grain boundary B2 particles makes them prone to becoming recrystallization nucleation sites, causing new recrystallized grains to form preferentially near B2 particles, which refines grains and improves recrystallization efficiency. This “particle-stimulated nucleation” mechanism is more significant when B2 particles have a moderate size (0.5–1 μm), while excessively fine (<50 nm) or coarse (>2 μm) B2 particles mainly play an obstructive role [19].

### 4.3. The Strengthening Mechanism

In order to further explore the strengthening mechanism of this steel, the contributions of solid solution strengthening, grain boundary strengthening, dislocation strengthening, and precipitation strengthening to the strength of the A900-3 and A900-15 samples are calculated. Specifically, solid solution strengthening comes from the alloying elements, grain boundary strengthening derives from the recrystallized grains, dislocation strengthening mainly occurs in the nonrecrystallization region, and precipitation strengthening originates from the B2 precipitates. The specific expression of total yield strength is as follows:

(1) $$\sigma_{YS}={\sigma_{0}+\sigma }_{s}+\sigma_{g}+\sigma_{d}+\sigma_{p}$$

where σ_0_ is the initial yield strength, σ_s_ is the stress of solid solution hardening, σ_g_ is the stress of grain boundary strengthening, σ_d_ is the stress of dislocation strengthening, and σ_p_ is the stress from precipitation strengthening.

(2) $$\sigma_{s}=97+187\;wt.\%C+20\;wt.\%Al+2.9\;wt.\%Ni+7\;wt.\%Cr-2\;wt.\%Mn$$

σ_s_ was estimated by Equation (2), through the individual solution hardening effect [37,38,39,40] and the mass percent of the alloying element.

(3) $$\sigma_{g}=k_{G}d^{-1/2}$$

σ_g_ is described by the Hall–Petch relationship (Equation (3)), where k_G_ is the Hall–Petch coefficient (461 MPa∙μm^1/2^ [41]), and d is the average austenite grain size.

(4) $$\sigma_{B2-\text{matrix}}=\left(0.538Gbf^{1/2}/d\right)ln\left(d/2b\right)$$

(5) $$\sigma_{B2-GB}=676+{1004d}^{-1/2}$$

σ_p_ was estimated based on the rule of mixture, where σ_B2-matrix_ is the strength contributed from B2 precipitates within the austenite matrix, and σ_B2-GB_ is the strength contributed from B2 precipitates at grain boundaries. σ_B2-matrix_ is calculated by Equation (4) [42], and σ_B2-GB_ is calculated by Equation (5) [43]. G (70 GPa) and b (0.26 nm) [44] are the shear modulus and the magnitude of the Burgers vector, respectively. f is the volume fraction of B2 precipitates, and d is the average equivalent diameter of B2 particles.

(6) $$\sigma_{d}=M\alpha Gb\rho^{1/2}$$

(7) $$\rho_{dis}=2\theta /\mu b$$

σ_d_ was calculated by Equation (6) [45], where M is the Taylor factor of austenite steel (3.06) [44], α is a constant for austenite metal (0.2) [46], and ρ is the dislocation density. The dislocation density ρ_dis_ was calculated by Equation (7) [47], where θ is the misorientation angle, and μ is the unite length (0.1 μm). The misorientation angle was estimated based on the KAM map, which gives a dislocation density of ~1.2 × 10^15^ m^−2^ in the unrecrystallized austenite region.

The calculated strength values of these strengthening mechanism contributions for the two samples are summarized in Table 2. It is evident that solid solution strengthening offers the greatest strength contribution, measuring 326 MPa for the A900-3 sample and 328 MPa for the A900-15 sample. The second contribution comes from grain boundary strengthening, which accounts for 227 MPa in the A900-3 sample and 289 MPa in the A900-15 sample. The A900-15 sample has a higher number of micron-sized recrystallized grains, resulting in a more pronounced grain boundary strengthening effect. The third mechanism is precipitation strengthening from the B2 phase, which contributes 167 MPa for the A900-3 sample and 126 MPa for the A900-15 sample. The lower Ni content and B2 volume fraction in the present steel result in a reduced strength contribution from B2 precipitate strengthening compared to other types of 5% Ni steels [19,32]. Dislocation strengthening is related to dislocation density in the unrecrystallized region. Thus, its effectiveness is limited when the volume fraction is small. In the A900-3 sample, the strength contribution from dislocation strengthening is 134 MPa. It is noteworthy that the calculated yield strength is lower than the experimental values because the strengthening effects of ferrite and nanoscale κ-carbides were not taken into account. This difference between the two values can be estimated to contribute a total strength of approximately 260 MPa from these two parts. Together, all these mechanisms enable the steel to achieve a significant synergistic strengthening effect.

### 4.4. A Comparison of Mechanical Properties

The studies on Fe-Mn-Al-C-(Ni) lightweight steels demonstrate that the specific tensile strength and specific yield strength can be significantly enhanced through microstructural design and thermomechanical processing. For instance, the dual-heterogeneous structured Fe-24Mn-11Al-0.9C-5Ni (wt.%) steel [25] achieved a yield strength of 1039 MPa and a tensile strength of 1300 MPa, accompanied by a total elongation of 39.3%. This performance was attributed to the bimodal grain size distributions of both austenite and B2 phases, which induce strong heterogeneous deformation-induced strengthening. Additionally, Fe-18Mn-8Al-1C-5Ni (wt.%) steel [48], optimized through annealing and aging to control B2 and κ-carbide precipitations, reached a yield strength of 1220 MPa, a tensile strength of 1423 MPa, and a total elongation of 35.8%, obtaining a balance of strength and ductility effectively. These findings collectively highlight that regulating the size, distribution, and volume fraction of B2 phases and κ-carbides, along with engineering heterogeneous microstructures, is crucial for achieving superior specific strength in lightweight steels.

**Figure 10:**
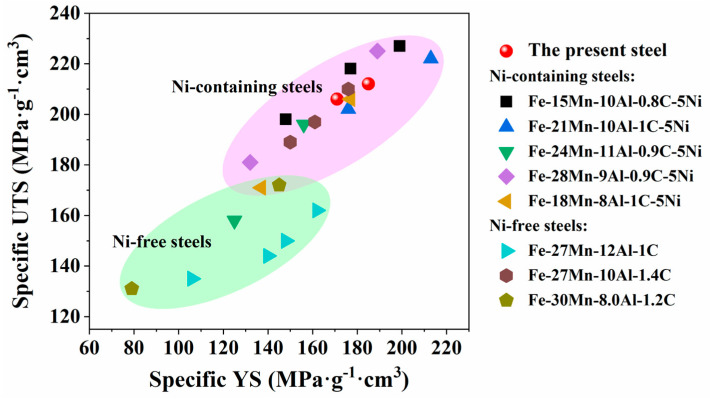
Specific UTS versus specific YS of present steel compared to other Ni-containing [17,19,25,48,49] steels and Ni-free steels [1,9,50].

Figure 10 clearly illustrates the specific ultimate tensile strength (UTS) versus specific yield strength (YS) of the present steel compared to other B2-strengthened steels and κ-strengthened steels. The data points within the green region represent Ni-free steels, which primarily enhance their strength through the precipitation strengthening of κ-carbides. The data points in the pink region correspond to Ni-containing steels, which demonstrate relatively higher specific yield and tensile strengths, as shown in this figure. These Ni-free steels exhibit relatively lower specific strength, indicating that reliance solely on κ-carbide strengthening is less effective. In addition to utilizing the combined precipitation strengthening of the B2 phase and κ-carbides, these Ni-containing steels benefit from composite strengthening mechanisms such as grain boundary strengthening and dislocation strengthening.

The red dots represent the steel studied in this research, which attains a high level of specific ultimate tensile strength (206~212 MPa·g^−1^·cm^3^) and specific yield strength (171~185 MPa·g^−1^·cm^3^) among Ni-containing steels, suggesting that the developed steel exhibits favorable performance in terms of lightweight design and high strength. Compared with other B2-strengthened steels, such as Fe-15Mn-10Al-0.8C-5Ni (wt.%) steel [17] with a specific ultimate tensile strength of 227 MPa·g^−1^·cm^3^ and Fe-21Mn-10Al-1C-5Ni (wt.%) steel [19] with a specific ultimate tensile strength of 213 MPa·g^−1^·cm^3^, the developed steel has slightly lower strength but still shows good performance. Compared with the κ-strengthened steels, such as Fe-27Mn-12Al-1C (wt.%) steel [1] with a specific yield strength of 162 MPa·g^−1^·cm^3^, the developed steel has a significant advantage in specific strength. This work establishes a crucial theoretical foundation for the design and optimization of high-strength lightweight steels. The combined approach of composite strengthening with optimized composition and processing parameters shows promising potential for further enhancing specific strength properties.

## 5. Conclusions

In summary, this study systematically investigates the microstructural evolution and mechanical properties of a novel Fe-Mn-Al-C-Ni-Cr lightweight steel with a lower Ni content (3.2 wt.%) and an appropriate Cr content (3.9 wt.%), compared with other Ni-containing steels. By optimizing the composition and regulating the microstructure, the steel achieves a high specific strength through the synergistic effects of multiple strengthening mechanisms. The following conclusions can be drawn:

(1) Annealing at 900 °C significantly influences the microstructure evolution of the steel. Short-time annealing results in a microstructure characterized by ultrafine recrystallized austenite grains (1.1 μm), fine B2 precipitates (0.18 μm in matrix and 0.4 μm at grain boundaries) with a relatively high volume fraction (9.5%), and uniformly dispersed nanoscale κ-carbides (<10 nm). Long-time annealing (15 min) leads to the coarsening of austenite grains (1.6 μm) and grain boundary B2 particles (0.9 μm), along with a reduction in B2 volume fraction (8.9%).
(2) The steel achieves an exceptional strength–ductility balance (YS: 1130~1218 MPa; UTS: 1360~1397 MPa; elongation: 18~23%). Quantitative strengthening analysis reveals dominant contributions from solid solution strengthening (326~328 MPa) and grain boundary strengthening (227~289 MPa), with additional precipitation hardening (126~167 MPa) from B2 phases and dislocation strengthening. The 260 MPa difference between the calculated and experimental yield stresses is attributed to the contributions of ferrite and κ-carbide strengthening.
(3) The developed steel exhibits excellent mechanical properties, achieving specific yield strengths of 171~185 MPa·g^−1^·cm^3^ and specific ultimate tensile strengths of 206~212 MPa·g^−1^·cm^3^. This superior performance is achieved through innovative compositional design and the optimization of the hot-rolling, cold-rolling, and annealing process to control recrystallization kinetics and precipitate distribution.
(4) This work provides a new strategy for developing low-Ni lightweight steels through Cr addition and microstructural optimization, enriching the understanding of multiple strengthening mechanisms in Fe-Mn-Al-C steels. The high specific strength of the developed steel can contribute to automotive lightweighting, reducing energy consumption and carbon emissions, which aligns with global sustainability goals.

## Figures and Tables

**Table 1:** Volume fraction (%) and size (μm) of recrystallized (RC) austenite and ferrite grains, and B2 particles within grain boundary (GB) and on austenite matrix.

Sample		RC		B2	
		Austenite	Ferrite	GB	Matrix
A900-3	Fraction (%)	51.7	6.8	4.5	5.0
	Size (μm)	1.1	2.7	0.41	0.18
A900-15	Fraction (%)	79.3	6.8	5.3	3.6
	Size (μm)	1.6	3.2	0.88	0.33

**Table 2:** The estimated values of solid solution strengthening (σ_s_), grain boundary strengthening (σ_g_), dislocation strengthening (σ_d_), and precipitation strengthening (σ_p_) to the total estimated (est.) yield strength versus the experimental (exp.) yield strength of the samples.

Sample	σ_0_ (MPa)	σ_s_ (MPa)	σ_g_ (MPa)	σ_p_ (MPa)	σ_d_ (MPa)	σ_YS_ (MPa) (Est.)	σ_YS_ (MPa) (Exp.)
A900-3	97	326	227	167	144	961	1218
A900-15	97	328	289	126	23	863	1130

## Data Availability

The original contributions presented in this study are included in the article. Further inquiries can be directed to the corresponding author.
